# Supplementary material for: Reconciling Mining with the Conservation of Cave Biodiversity: A Quantitative Baseline to Help Establish Conservation Priorities
Source: PLoS One. 2016 Dec 20;11(12):e0168348. doi: 10.1371/journal.pone.0168348 (PMC5173368; doi:10.1371/journal.pone.0168348)
Supplement: S1 Dataset — (ZIP) [file pone.0168348.s002.zip › Taxa/Serra Norte/SN_2013/N5S/Lista N5S-32.pdf]

CAVIDADE N5S-0032

Classe	Ordem	Fam/Outros	Gên/Outros	Espécie	S-U	U-S	Zon.
Arachnida	Acari	Macronyssidae		sp.1		X	P
Arachnida	Acari	Rhagidiidae		sp.1		X	P
Arachnida	Amblypygi	Phrynidae	*Heterophrynus*	*longicornis*	2	1	E/P
Arachnida	Araneae	Drymusidae		jovem		X	P
Arachnida	Araneae	Mysmenidae	*Microdipoena*	sp.1	X		E
Arachnida	Araneae	Ochyroceratidae	*Speocera*	sp.1	X	X	P
Arachnida	Araneae	Pholcidae	*Leptopholcus*	sp.1	X		E
Arachnida	Araneae	Pholcidae	*Mesabolivar*	*eberhard*	X	X	E
Arachnida	Araneae	Pholcidae	Ninetinae	sp.1	X		E
Arachnida	Araneae	Salticidae		jovem	X		E
Arachnida	Araneae	Theraphosidae		jovem	2	10	E/P
Arachnida	Araneae	Theridiosomatidae	*Plato*	sp.1	X		E/P
Arachnida	Opiliones	Escadabiidae		sp.1		X	E
Arachnida	Opiliones	Gonyleptidae		jovem	X		E
Arachnida	Opiliones	Sclerosomatidae	*Prionostema*	sp.1	X	X	E
Arachnida	Opiliones	Stygnidae		jovem		2	E/P
Arachnida	Pseudoscorpiones	Chernetidae	*Spelaeochernes*	sp.1	X	X	E/P
Arachnida	Pseudoscorpiones	Chthoniidae	*Pseudochthonius*	sp.1	X		E
Diplopoda	Polydesmida	Pyrgodesmidae		sp.1		X	E
Entognatha	Collembola	Paronellidae		sp.1		X	E
Entognatha	Collembola	Paronellidae		sp.4	X		E
Entognatha	Diplura	Campodeidae		sp.1	X	X	E
Insecta	Blattodea			jovem	1	1	P
Insecta	Coleoptera	Scydmaenidae		sp.11	X		P
Insecta	Coleoptera	Staphylinidae		sp.66		X	E
Insecta	Coleoptera	Tenebrionidae		jovem	X		P
Insecta	Dermaptera	Pygidicranidae		sp.1		X	P
Insecta	Diptera	Cecidomyiidae	Cecidomyiinae	sp.		X	E
Insecta	Diptera	Ceratopogonidae		jovem	X		E
Insecta	Diptera	Phoridae	Metopininae	sp.	X		P
Insecta	Diptera	Psychodidae	*Sciopemyia*	*sordellii*	X	X	E/P
Insecta	Diptera	Tipulidae	Tipulinae	sp.	X		E
Insecta	Heteroptera	Cydnidae	*Pangaeus*	sp.1		X	E
Insecta	Heteroptera	Lygaeidae	Rhyparochrominae	sp.2	X		E
Insecta	Heteroptera	Reduviidae	Reduviinae	jovem	4	2	E/P
Insecta	Homoptera	Cixiidae		jovem	X	X	E/P
Insecta	Hymenoptera	Cabronidae		sp.1	X		E
Insecta	Isoptera	Termitidae	*Cortaritermes*	*silvestrii*	X	X	E/P
Insecta	Lepidoptera	Noctuoidea		sp.12		3	P
Insecta	Neuroptera	Myrmeleontidae		jovem	1	1	E
Insecta	Orthoptera	Phalangopsidae	*Paraclodes*	sp.	9		E
Insecta	Orthoptera	Phalangopsidae	*Phalangopsis*	sp.	36	11	P
Insecta	Psocoptera	Epipsocidae	*Epipsocus*	sp.1	X	X	E
Insecta	Thysanura	Nicoletiidae		sp.1		X	E/P
Gastropoda	Pulmonata	Systrophiidae	*Happia*	sp.		X	E
Amphibia	Anura	Strabomantidae	*Pristimantis*	cf. *fenestratus*	1		P
Amphibia	Anura			sp.4	1		P
Mammalia	Chiroptera	Phyllostomidae	*Anoura*	sp.		4	P
Mammalia	Chiroptera	Phyllostomidae	*Carollia*	*perspicillata*	3	2	P

Mammalia	Chiroptera	Phyllostomidae	Glossophaginae	sp.	6	10	P
Mammalia	Chiroptera	Phyllostomidae	*Micronycteris*	sp.	6	15	P
